# A Roadmap for the Rational Use of Biomarkers in Oral Disease Screening

**DOI:** 10.3390/biom14070787

**Published:** 2024-07-01

**Authors:** Nicola Cirillo

**Affiliations:** 1 Faculty of Medicine, Dentistry and Health Sciences, The University of Melbourne, 720 Swanston Street, Carlton, VIC 3053, Australia; nicola.cirillo@unimelb.edu.au; 2 School of Dentistry, University of Jordan, Amman 11733, Jordan; 3 CoTreatAI, CoTreat Pty Ltd., Melbourne, VIC 3000, Australia

**Keywords:** biomarkers, oral cancer, periodontitis, dental caries, cardiovascular disease, screening

## Abstract

Oral health has witnessed a significant transformation with the integration of biomarkers in early-diagnostic processes. This article briefly reviews the types of biomarkers used in the screening and early detection of oral diseases, particularly oral cancer, periodontal diseases, and dental caries, with an emphasis on molecular biomarkers. While the advent of these biomarkers may represent a leap forward in oral healthcare, it also opens the door to potential overtesting, overdiagnosis, and overtreatment. To inform the selection of novel biomarkers and ensure their rational use in screening tests, it is imperative to consider some key characteristics, which are specific to the biomarker (e.g., surrogate biomarkers should reliably reflect the primary health outcome), to the test (e.g., sensitivity and specificity must be balanced based on the disease of interest), and to the disease (e.g., the efficacy of treatment should improve when the condition is diagnosed earlier). For systemic conditions associated with oral diseases, researchers should be extremely cautious when determining who is “at risk”, particularly when such risk is small, non-existent, or inconsequent. This framework aims to ensure that advancements in oral health diagnostics translate into genuine improvements in patient care and well-being.

## 1. Introduction

Medical care, including oral health care, has witnessed a significant transformation with the integration of biomarkers in diagnostic processes. Biomarkers, which are broadly defined as “characteristics that are measured as indicators of normal biological processes, pathogenic processes or responses to an exposure or intervention” [1], are increasingly being utilized for early detection and management of oral diseases [2]. This evolution is partly due to the rapid advancement in diagnostic technologies, which promises a more personalized and preventive approach to dental care. Although historically associated with physical traits or physiological metrics, the term now commonly refers to molecular biomarkers [3]. These exist in various forms, leading to the adoption of a variety of strategies for their development. The selection and use of molecular biomarkers for screening of oral diseases is the focus of this article.

Biomarkers used for screening and early detection in oral health serve primarily as indicators of pathological processes or for risk-stratification algorithms. Salivary biomarkers, for instance, have gained attention for their non-invasive collection methods and potential in diagnosing a range of oral conditions, from periodontal disease to oral cancer [4,5]. Similarly, blood biomarkers have been explored for their role in identifying systemic conditions that affect oral health, such as diabetes and cardiovascular diseases, which have implications for periodontal health [6]. Tissue biomarkers, on the other hand, are pivotal for the histological analysis of oral lesions and malignancies in follow-up tests, offering insights into tumor biology and the potential for malignant progression [7].

While the advent of biomarkers represents a leap forward in oral healthcare, it also opens the door to potential overtesting and overdiagnosis (and possibly overtreatment). The enthusiasm for these novel diagnostics must be tempered with an understanding of their limitations and the potential for false positives and overinterpretation of results. For example, the specificity and sensitivity of salivary biomarkers in detecting oral cancer are still suboptimal, with concerns about the accuracy of the tests and the high risk of bias raised in recent meta-analyses [8,9].

The rise of diagnostic technologies also reflects a broader trend in healthcare towards more technologically advanced, yet potentially excessive, medical interventions [10]. This is particularly relevant as most adjuncts and biomarkers do not add to the diagnostic accuracy [11]. Hence, the shift towards high-tech diagnostics, while offering substantial benefits in principle, also raises questions about the cost-effectiveness and the potential for unnecessary treatments. Studies have shown that an increase in diagnostic testing may not equate with improved patient outcomes, and, in some cases, it may lead to unnecessary anxiety and medical procedures [12].

As with any new medical technology, it is important to leverage the benefits of the use of biomarkers but also to appreciate the limitations and potential risks associated with these tools. This article discusses the different types of biomarkers that are potentially useful in dental practice, particularly those suitable for screening and early detection of relevant oral diseases. Further, it aims to critically explore the delicate balance between benefits and limitations and to examine key characteristics that must be considered when utilizing biomarkers in a clinical practice. The ultimate goal of this roadmap is to inform a rational selection of molecular biomarkers, thus ensuring that their use translates into genuine improvements in patient care and well-being.

## 2. Types of Biomarkers and Their Use in Orofacial Diseases

Biomarkers encompass a diverse range of biological indicators, each offering unique insights into the health status of an individual. Biomarkers can be classified in different ways, for example, based on their nature (e.g., clinical, imaging, and molecular), origin (e.g., tissue, blood, and saliva), or use in various clinical and research contexts (e.g., diagnostic, prognostic, and monitoring). Each type of biomarker may serve a unique purpose in healthcare, by aiding in the diagnosis, treatment decision-making, monitoring of disease progression, and assessment of treatment efficacy and safety. To overcome the ambiguity surrounding definitions, which has led to delays or even halts in the advancement of effective diagnostic and therapeutic technologies, biomarker definitions were established in a joint FDA-NIH resource—the “Biomarkers, EndpointS, and other Tools” (BEST) [1]. Importantly, this document does not include a definition of the screening biomarker, as it was assumed that any biomarker could be used for screening purposes. However, there are clear intrinsic differences between the screening and other biomarkers; hence, this type of biomarker has been considered as a category of its own for this review (Table 1).

Screening biomarkers are typically employed in a broader population to detect early signs of a disease before symptoms appear. Although diagnostic, prognostic, predictive, and risk biomarkers are often used interchangeably in certain contexts, there are differences. For example, while screening biomarkers aim to identify those who may need further diagnostic testing, diagnostic biomarkers are used to confirm or rule out the presence of a disease in individuals who are suspected of having the disease, typically based on screening results or symptoms. Screening biomarkers are sometimes used with the aim of assessing the risk or likelihood of developing a disease, allowing for early preventative measures, in which case they may overlap with risk/susceptibility biomarkers. Prognostic and predictive biomarkers are also commonly used as synonyms; however, their distinction is important when assessing likely disease outcomes. Prognostic biomarkers are associated with differential disease outcomes, whereas predictive biomarkers discriminate between those who will and will not respond to therapy [13].

**Table 1:** Types of biomarkers based on their use in clinical and research contexts.

Type	Use	Example	Ref
Screening	Used to identify the possibility of a disease in an asymptomatic individual	Measurement of blood pressure, blood glucose, cholesterol, body mass index in primary care dental practice	[14]
Diagnostic	Used to identify or confirm the presence of a disease or condition	Elevated levels of anti-SSA and/or anti-SSB antibodies in Sjogren’s Syndrome	[15]
Prognostic	Predict how a disease will progress over time, regardless of the treatment.	Immune-desert phenotype on histology predicts poor prognosis in tongue OSCC.	[16]
Predictive	Helps predict how well a patient will respond to a specific treatment or therapy.	Periodontal pathogen loads to predict progressive periodontitis in the subsequent supportive periodontal therapy phase	[17]
Pharmacodynamic	These are used to show that a drug has reached its intended target in the body and is having its desired effect.	Reduction of prostaglandin E(2) levels in gingival crevicular fluid following adjunctive use of flurbiprofen during periodontal therapy	[18]
Safety	These indicate the potential toxic effects of a treatment or drug, helping to assess its safety profile	Urinary concentration of fluoride to quantify fluoride exposure and monitor toxicity	[19]
Monitoring	Used to monitor the status of a disease or the effects of treatment over time	Measurements of serum autoantibody titers in Pemphigus Vulgaris to monitor disease progression and for treatment guidance	[20]
Risk/Susceptibility	Indicates the likelihood of a patient developing a disease or condition, often used in the context of preventive medicine.	Serial measurement of salivary SMR-3B and mucin-7 for dynamic surveillance of changes in high-risk status of early childhood caries	[21]

The molecular biomarkers most commonly used in oral health research can be broadly categorized into three types: salivary, blood, and tissue biomarkers. Each type has the potential to play a critical role in diagnosing and monitoring oral diseases, offering the potential for early intervention and personalized treatment strategies.

### 2.1. Salivary Biomarkers

Saliva, as a diagnostic fluid, offers a wealth of information about both oral and systemic health. It is a complex biological fluid mainly composed of secretions from the salivary glands and upper respiratory tract, gingival crevicular fluid, plasma exudates, cell debris, and components of microorganisms. There is a broad spectrum of molecules in saliva, such as inorganic ions, DNA, mRNA, miRNA, proteins (cytokines, enzymes, chemokines, immunoglobulin, etc.), and metabolites, whose variations in concentration can potentially be used as biomarkers to detect oral diseases at an early stage or to monitor the response to a therapeutic management plan (reviewed in [22]). The non-invasive nature of saliva collection makes it an ideal medium for regular monitoring of oral health conditions. For instance, specific salivary enzymes have been linked to periodontal disease, while changes in salivary pH and composition can indicate caries risk. Additionally, the presence of certain molecules in saliva (often referred to as salivaomics) has been studied as a potential biomarker for oral cancers [23].

A recent scoping review identified and analyzed 92 different biomarkers in human saliva to find out if specific molecules had the potential for personalized oral healthcare and precision dentistry [24]. The authors concluded that, currently, no consensus exists on which biomarkers should be used for what specific scientific target(s) and with which intention. Therefore, no clear recommendations can be given related to the specific salivary biomarkers associated with personalized oral healthcare principles.

### 2.2. Blood Biomarkers

Blood biomarkers play a pivotal role in understanding the systemic implications of oral diseases and, together with salivary biomarkers, have informed the development of the so-called “liquid biopsy” [25]. Research has established connections between periodontal disease and systemic conditions like cardiovascular diseases and diabetes, with inflammatory markers in blood being common indicators [26]. C-reactive protein (CRP), for example, is a well-known inflammatory marker that has been associated not only with periodontal disease and cardiovascular risk but also with peri-implantitis [27].

Together with saliva, blood is probably the best-characterized tissue for dental applications of liquid biopsy. These analyses employ multiple biomolecular sources including circulating tumor DNA (ctDNA), circulating tumor cells (CTCs), and exosomes (that contain DNA fragments) to detect genetic biomarkers that can predict, disclose, and/or monitor cancers [28].

### 2.3. Tissue Biomarkers

Tissue analysis, particularly in the context of oral cancer and precancer, has become increasingly important. Biomarkers in tissue samples, such as genetic mutations or protein expressions, can provide information about the aggressiveness of a tumor, its potential response to treatment, and the likelihood of recurrence [29]. For example, the overexpression of certain proteins, like p53, has been associated with oral squamous cell carcinoma and can be used as a prognostic indicator [30]. However, tissue biomarkers are not well suited to screening, as they typically derive from specimens of diseased tissue. An application of tissue biomarkers in dentistry is provided by oral exfoliative cytology, which has been suggested not only for oncological screening [31] but also for the evaluation of diabetes [32] and iron overload [33].

It is worth noting that the expression of the same biomarkers from different tissues may vary; for example, a recent meta-analysis has shown a significant decrease in SOD levels in both erythrocyte samples and tissue samples in individuals with oral cancer (OSCC) compared to the normal control group, whereas the opposite was true in salivary samples, which demonstrated a significant increase in SOD levels in cancer patients [34]. These variations underscore the importance of considering the tissue-specific context when interpreting biomarker data.

## 3. Molecular Biomarkers for Screening and Early Detection in Dentistry

A major difference between a diagnostic biomarker and one used for screening is that the latter is utilized in healthy patients, or at least to those without signs or symptoms of the illness being screened for. Hence, a screening test may be defined as a type of medical examination or procedure carried out on people in a specific, symptom-free group to determine their likelihood of having a certain disease [35]. A major objective of most screening tests is to diagnose the condition of interest early (before symptoms arise) and by so doing reduce the morbidity or mortality in the population group being screened. As routine dental “check-ups” occur regardless of specific symptoms, this practice may be considered as opportunistic screening.

A number of screening tools have been developed for dental use, from fluorescence-based devices for early caries detection [36] to artificial intelligence-guided image analysis for oral cancer and precancer [37]. In parallel, research has revealed numerous connections between biological measurements and disease models across various levels, from the subcellular to the intact organism [38]. This expanding capacity to measure differences at a molecular level has resulted in a wealth of potential molecular biomarkers for disease states, with the aim of improving the accuracy of current diagnostic standards and procedures. Here, we explore the usability of biomarkers in oral diseases featuring different clinical and epidemiological characteristics: oral cancer, a relatively rare but fatal condition; periodontitis, a common but usually not severe pathology overall, yet harboring the risk of serious systemic diseases; and dental caries, one of the world’s most common conditions, but with limited medical significance in terms of morbidity and mortality (Table 2).

### 3.1. Oral Cancer

In the last 15 years, there has been an explosion in experiments on biomarkers for identifying OSCC and/or predicting the malignant transformation of precursor lesions using complex computational methods [53]. As population-wide screening is likely inefficient given the relatively low prevalence of oral cancer, risk prediction models have been proposed to target screening to those at the highest risk or select individuals, for example, those with precursor lesions or OPMDs [54]. Predictive models have used a wide range of risk factors, including clinical, genetic, and blood-based biomarkers, in addition to demographics and lifestyle risk factors [55]. Among the molecular biomarkers tested in these models, arsenic, selenium, and cerium levels, as well as single nucleotide polymorphisms (SNPs), were included in a recent systematic review [9], but many others have been assessed as independent predictive tools, chiefly TP53, CDKN2A, NOTCH1 and aneuploidy in tissues [56,57,58].

Saliva has gained attention as a non-invasive, easy-to-collect source of potential biomarkers useful in the early detection of disease states. Currently, its main limitation is that, while the expression of many salivary biomarkers is significantly altered in OPMDs and OSCC as compared to healthy controls [59], specificity and risk stratification/prognostic potential remain an issue. Our group developed a high-risk dysregulation signature based on salivary and tissue miRNA expression (miR-24; miR-21; miR-31; miR-99a; let-7c; miR-100; and miR-125b, in various combinations) that was able to identify OSCC patients with 86.8% sensitivity and 81.5% specificity [60,61]. Systematic reviews assessing the expression of circulating (miRNA27, miRNA 21, and miRNA 155) [62] and tissue-based miRNAs [63] have reported diagnostic and prognostic significance, respectively, in OSCC patients. A large number of microRNAs from liquid biopsy samples (whole blood, serum, plasma, and saliva) have been suggested as potential markers of premalignancy and/or malignancy [64]. While these results are encouraging, specificity is still suboptimal when applied to a large population such as OPMD patients, and therefore the risk of overdiagnosis is high. Overall, the utility of miRNAs as biomarkers in predicting cancer progression in patients with OPMDs is still equivocal [65].

With regard to markers that could be suitable for population screening, recent meta-analyses assessing salivary biomarkers for early detection of OSCC concluded that chemerin and MMP-9 are the most promising molecules [66], with high sensitivity (0.94) and a balanced accuracy of 0.93 [9]. There is also evidence that IL-6, IL-8, and TNF-α are useful inflammatory cytokines for the early diagnosis of OSCC [39,67]. None of these tools have been demonstrated to be useful in clinical settings to date.

### 3.2. Periodontal Disease and Oral-Systemic Health

The onset of periodontal disease is intimately related to the composition of the oral microbiota, and both topics have gained considerable attention in the study of potential oral-systemic links. In particular, patients diagnosed with periodontitis have been found to be at increased risk of diabetes, cardiovascular and respiratory diseases, and many other health conditions including COVID-19 complications and brain disease [26]. Consequently, screening for periodontitis could have a far-reaching impact on general health. To be sure, since periodontitis is bi-directionally associated with several systemic diseases, the aim of screening would be twofold. On the one hand, diagnosis of periodontitis may serve as a gateway for “risk assessment” and/or early detection of other relevant medical conditions—for example, undiagnosed diabetes via HbA1c levels in gingival crevicular blood [68]; on the other, medical professionals in a non-dental setting may facilitate the detection of periodontitis in medically compromised patients using appropriate screening tools [69]. It is not surprising, therefore, that a multitude of (screening) tests have been developed to assess comorbidities as well as quality of life in patients with periodontitis. This risk-based approach also applies to the so-called “predictive” periodontitis, which promises to identify tailor-made treatments for individual patients [70].

While single biomarkers display suboptimal performance [71], a recent meta-analysis found six salivary combinations with acceptable sensitivity and specificity (median sensitivity/specificity values are reported in parentheses): IL-6 with MMP-8 (86.2%/80.5%); IL-1β with IL-6 (83.0%/83.7%); IL-1β with MMP-8 (82.7%/80.8%); MIP-1α with MMP-8 (71.0%/75.6%); IL-1β, IL-6, and MMP-8 (81.8%/84.3%); and IL-1β, IL-6, MIP-1α, and MMP-8 (76.6%/79.7%) [45].

There are, however, three important caveats to the translation of periodontal biomarker discovery into clinical practice. First, the data that inform this risk-based approach are drawn from observational studies that are often flawed because what is considered a “risk” may, in fact, be the residual effect of confounding variables (for example, smoking is a risk factor for both periodontitis and cardiovascular disease, hence a correlation exists between these two; this applies to many other confounders), and when a statistically significant risk does exist, it is often so small as to be not clinically significant [72]. The second point is that there is little value in finding a risk if the risk cannot be lowered. Hence, the strength of the oral–systemic connection and the importance of identifying systemic diseases early, in the context of a dental setting, relies somewhat on the expectation that control of periodontitis improves patient outcomes for the associated condition. However, whether periodontal treatment can in fact do so remains uncertain. The DIAPERIO randomized controlled trial found that periodontal treatment had no clinical effect on glycemic control [73], consistent with the results of a previous randomized trial on non-surgical periodontal treatment [74]. The effect of intensive periodontal treatment on HbA1c levels became statistically significant, albeit with a meager effect size, only when diabetic patients with moderate-to-severe periodontitis were considered [75]. The third caveat feeds into current medical controversies on the clinical significance of conditions like “prediabetes” or “prehypertension”. For example, a small study from Victoria found that a large proportion of patients with periodontitis had undiagnosed prediabetes, hypertension, hypercholesterolemia, and elevated BMI levels [76]. However, there is a wealth of research supporting the notion that hypercholesterolemia, overweight, and prediabetes (total cholesterol/TC ≥ 5.5, body mass index/BMI above 25, and HbA1c of ≥5.7, respectively, in the study) are in fact not dangerous to human health when the “abnormal” values are at the mild end of the spectrum. For example, a BMI between 25 and 30 (which is technically labeled as overweight) is associated with a lower all-cause mortality compared to a normal BMI [77]. In patients who have had a stroke, being overweight (but not obese) is even associated with reduced mortality and better functional outcomes [78]. Therefore, we should be extremely cautious when attempting to establish who is “at risk”, particularly when such risk is small or non-existent.

### 3.3. Dental Caries

Tooth decay is likely the most common disease of human beings in modern society. For decades, dentists have relied on a combination of visual–tactile and radiographic examination to identify dental caries accurately [79]. Recent systematic reviews have shown that visual inspection has a higher sensitivity (i.e., the proportion of decayed teeth correctly identified by the test) for detecting early caries and enamel lesions compared to bite-wings, especially non-cavitated lesions that may be missed by traditional radiographs [80]. The ability to accurately diagnose caries by visual examination has salient implications for image analysis and tele-dentistry and, accordingly, a myriad of artificial intelligence-based detection tools have been developed [81].

Early detection offers an opportunity to stabilize disease or even remineralize the tooth surface, thus preventing a lifelong cycle of restoration. Among the molecular biomarkers, mucin, histatin, proline-rich proteins (PRP), lactoperoxidase, C-reactive proteins, cathelicidin (LL-37), immunoglobulin (IgA), albumin, statherin, salivary total protein, SOD, copper, zinc, proteinase 3 (PR3), alpha-amylase, and carbonic anhydrase have been assessed [50] without conclusive results. A useful application of molecular biomarkers could be the assessment of caries activity, because this determination directly influences clinical decision-making, e.g., to provide restorative treatment vs. non-operative management [82]. Commercial kits that measure surrogate endpoints such as salivary flow rate, salivary pH, and buffering capacity are available for chairside clinical tests (reviewed in [83]) but their use in caries risk assessment is not widely accepted [84].

While different diagnostic means have been tested as adjuncts to clinical examination [85], none of these tools has convincingly shown improvements in detection rates, not even for enamel caries [86]. Furthermore, it has long been known that not all lesions progress to cavitation, and the real challenge is to determine which white-spot lesions will progress. Remarkably, incipient lesions confined to the enamel may never involve the underlying layers, and even advanced caries (ICDAS 3 and 4) will not progress to cavitation over 4 years in a sizeable number of patients [87]. Therefore, the value of molecular biomarkers as screening tools for caries early detection is of questionable public health impact.

## 4. A Roadmap to Wisely Choosing Biomarkers for Oral Disease Screening

While increasingly sensitive screening and diagnostic tests enable the detection of many potentially severe chronic diseases at the earliest stages, they concomitantly expand the disease reservoir of subclinical conditions from which overdiagnosis occurs [88]. It is therefore important to take into consideration the key features of a molecular biomarker used for the screening of oral diseases.

### 4.1. Biomarker-Dependent Features

#### 4.1.1. Surrogate Markers for the Disease Should Be Validated and Meaningful

It is often inefficient, impractical, or unsafe to use the diagnostic “gold standard” for screening; hence, a surrogate marker (or endpoint in clinical research) is chosen. While this is a substitute measure, it is believed to consistently reflect or predict the primary outcome. Determination of the circulating levels of prostate-specific antigen (PSA), rather than the more invasive procedure of histological detection of prostate cancer through biopsy, is a typical surrogate biomarker used for screening [89]. In dentistry, tentative examples might include inflammatory biomarkers such as IL-6 and CRP for early detection of periodontitis [90] and altered tissue fluorescence or DNA content in cancer tissues that can be visualized through appropriate tools [91]. The location of mouth diseases makes the use of “proximal” oral biofluids such as saliva credible surrogates harboring tissue-derived molecular alterations.

The most prevalent and critical mistake in assessing biomarkers is assuming that a correlation between the biomarker’s level and a clinical outcome automatically deems the biomarker a reliable surrogate [13]. To qualify as a surrogate, a biomarker must not only show a correlation with the outcome but also demonstrate that changes in the biomarker correspond to changes in the clinical outcome. This concept relies on statistical inference, which requires observations across multiple conditions and therapies that affect the biomarker. Unfortunately, the vast majority of biomarkers are not valid surrogates, and even when a surrogate is validated, its reliability is limited to a specific context for use.

#### 4.1.2. An Ideal Biomarker Should Be Easy to Measure

The practicality of measuring a biomarker is vital for its widespread adoption and utility in clinical settings. Salivary biomarkers (and others from proximal fluids such as crevicular fluid) are advantageous in this regard because their collection is relatively simple and can be performed without specialized training or equipment. Additionally, saliva samples can be stored and transported more easily than other biological samples. On the other hand, tests relying on salivary biomarkers require complex manufacturing.

#### 4.1.3. Biomarkers Should Be Consistent across Genders and Ethnic Groups

Screening biomarkers should demonstrate consistent effectiveness in detecting or predicting the presence of a particular condition across different demographic groups, including genders and ethnicities. If a biomarker is effective in one group but not in another, it will not be applicable and generalizable to diverse populations. Consistency ensures that screening protocols and diagnostic criteria can be uniformly applied across different demographic groups without compromising accuracy or reliability.

### 4.2. Test-Dependent Features

#### 4.2.1. The Cost of the Screening Test and Follow-Up Tests Should Be Relatively Low

The success of screening programs depends on their cost effectiveness. The incremental cost-effectiveness ratio (ICER) threshold is typically used to guide decisions on screening programs by government and healthcare providers [92]. If screening costs are paid by the recipient, individuals are more willing to undergo screening when tests are affordable, leading to higher screening coverage and increased early detection rates. Similarly, the follow-up procedures can impose a significant financial burden on individuals, particularly those without adequate insurance coverage. Affordable follow-up tests alleviate this burden, reducing out-of-pocket expenses for individuals and families.

#### 4.2.2. The Test Should Be Safe and Simple

Measuring a biomarker should not pose any risk or harm to the individual being tested, and this is particularly important for screening tests that are administered to a potentially large portion of the population. For example, X-rays for dental radiographs do harbor some biological risks [93], and hence less “invasive” tests should be preferred whenever possible—for example, visual observation for dental check-ups with opportunistic screening of caries. Furthermore, tests with a smooth learning curve are more advantageous compared to those that require substantial operator training (e.g., fluorescence tools for oral cancer screening). For this reason, the assessment of molecular biomarkers in proximal biofluids has the potential to be a safe and simple method for screening.

#### 4.2.3. The Test Should Ideally Be Sensitive, Specific, and Have a High Predictive Value

For diagnostic tests, high sensitivity indicates that the test rarely misses individuals who truly have the disease (few false negatives), whereas high specificity indicates that the test rarely incorrectly identifies individuals as having the disease when they do not (few false positives). While high sensitivity and specificity are essential characteristics of diagnostic tests, there is a common perception that the accuracy of screening tests may be acceptable at lower thresholds. This is because screening is used to identify individuals who may have a higher *risk* of developing a particular disease or condition, often before symptoms are present. In the context of screening, therefore, sensitivity refers to the ability of the test to correctly identify individuals who are at risk of developing the disease. While specificity in screening tests remains important to minimize unnecessary follow-up testing or interventions in individuals who are not at risk of developing the disease, in some screening scenarios, specificity may be sacrificed to maximize sensitivity, ensuring that fewer cases of the disease are missed. This, however, increases the number of false positives and the overdiagnosis rate and possibly results in poor commercial performance. For example, while some point-of-care devices assessing protein biomarkers, such as MMP-8 [94], have been marketed for periodontitis, most have disappeared very quickly due to their low specificity [95]. With the tremendous advances in molecular techniques developed in the last 20 years, there is an urge to give a higher emphasis to the specificity of screening tests, particularly for universal and population-based screening.

### 4.3. Disease-Dependent Features

#### 4.3.1. Epidemiology of the Disease for Which Screening Is Envisaged

A key element that informs the acceptability of screening test performance is the epidemiology of the disease, particularly in terms of the prevalence and morbidity/mortality in the target population. Specifically, acceptable trade-offs among performance characteristics such as sensitivity, specificity, and positive and negative predictive values (PPV and NPV) will vary depending on the potential harm associated with false positive and false negative results. For instance, when a test is employed for screening asymptomatic individuals in a population where the prevalence of the target disease is low, priority should typically be given to tests with a high specificity and PPV. This helps to minimize the occurrence of false positive results, which could lead to unnecessary medical interventions and psychological distress. In this regard, one should be mindful that retrospective case-control methods artificially inflate prevalence and thus make it easier to overestimate efficiency in biomarker disease detection by inflating PPV estimates [96]. When a screening test targets individuals who have a high risk of a life-threatening disease with established clinical benefits from early intervention, greater importance should be placed on the test’s sensitivity and NPV. These performance characteristics are often at odds, which may explain the absence of reliable screening tools for oral cancer. It is reasonable to propose that acceptable sensitivity may be lower for common conditions, but not for rarer diseases, whereas specificity must be higher for diseases with significant morbidity or mortality.

#### 4.3.2. Indication for Treatment

There should be a proven recommended treatment that favorably modifies the outcome of the disease targeted by the screening test. Screening might not provide any real additional value if, for instance, no follow-up treatment is available [97]. Similarly, it would be inefficient to screen a population for a disease for which there is no recommendation for treatment. For example, detection through screening of oral mucosal disease with little or no malignant potential is of limited value from a public health perspective, and likely inefficient. This is a real problem in oral cancer screening because many molecular biomarkers are significantly altered in both OSCC and OPMDs and/or fail to reliably predict the risk of transformation of OPMDs. In caries research, consensus guidelines do not recommend any treatment for incipient inactive caries (ICDAS 1), therefore their detection in low-risk patients (e.g., with good oral hygiene) is likely inefficient.

#### 4.3.3. Effectiveness of Early Treatment

The goal of screening is to detect diseases at an early stage when treatment may be more effective, ultimately reducing morbidity and mortality. This implies that treatment must be shown to improve patient outcomes when the disease is diagnosed early. Unfortunately, this is not easy to demonstrate, as most studies focusing on screening and early detection use biased surrogate measures (e.g., 5-year survival in oral cancer screening and length of tooth preservation in periodontal treatment). Hence, it is imperative that early detection improves the outcome of the disease targeted by screening, rather than simply modifying the surrogate biomarker.

Given that the effectiveness of early detection on treatment outcomes is central for demonstrating the rationale of screening, the development of molecular biomarkers for screening should first consider whether this principle is satisfied for the disease of interest.

In summary, consideration of disease-dependent features should be made when approaching the study of a novel biomarker/test: sensitivity of screening tests should be higher in diseases with significant morbidity or mortality (oral cancer > periodontal diseases > dental caries) and in high-risk populations; higher sensitivity should be the aim when screening disease with low prevalence. It is proposed that a workable guide could be 1. high specificity and sensitivity (>90%) for oral cancer; 2. high sensitivity (>85%) but lower specificity (>75%) for periodontitis; and 3. moderate (>75%) sensitivity and specificity for caries detection screening tests. In addition, the potential to improve treatment through early detection must guide a rational selection of appropriate disease-specific molecular biomarkers.

## 5. How Non-Rational Screening Can Do More Harm than Good

Screening is one of the cornerstones of preventive medicine. Its aim is to detect disease in its early and pre-symptomatic stages. With suitable treatment, screening can lead to disease eradication or prevention for individuals identified as at-risk. The basic assumption behind this concept is that treating diseases at an early stage may be simpler and less costly, making screening strategies potentially beneficial investments for healthcare systems [97,98]. However, the efficacy of screening is frequently gauged by improvements in surrogate markers that may not accurately represent the true health outcomes. In one example, a recent meta-analysis found that there is no extension of lifetime with common cancer screening tests [99]. On the other hand, the increasing use of advanced screening technologies, particularly in the context of prevalent chronic diseases, has resulted in the early detection of a greater number of conditions during their preclinical stage, i.e., prior to the onset of symptoms, even though these abnormalities may never become clinically relevant [88]. This has introduced the epidemiological concept of overdiagnosis, which refers to the identification of a condition that would have remained clinically silent throughout the patient’s lifetime had it not been detected [100].

In this context, the rational choice of a biomarker is central as it can mitigate the shortcomings of population screening, such as overestimation of the disease risk and false alarms.

### 5.1. Overestimation of Disease Risk

Screening tests may identify individuals as “at risk” based on certain biomarkers, even if they are unlikely to develop the disease in question. This can lead to overestimation of disease risk and unnecessary interventions for individuals who may not benefit from them. There are several examples of this side effect as a consequence of the selection of unsuitable biomarkers in dentistry [101,102]. In a notable example, observational studies linking periodontal and systemic health have mostly relied on the correlation between periodontitis and surrogate molecular biomarkers of systemic diseases, rather than true health outcomes. The most recent meta-analysis [103] has suggested that there is a bi-directional link between periodontal disease and dyslipidemia (TC, triglycerides (TGs), low-density lipoprotein (LDL), and high-density lipoprotein (HDL)). To be sure, blood lipids are commonly used as surrogate markers of cardiovascular health; however, they do not necessarily predict valid health outcomes; e.g., all-cause mortality is lower for individuals older than 50 years with TC or LDL-C above the recommended levels [104]. Furthermore, the effect size of a lipid-level increase in periodontal patients is very modest overall, as is the risk increase for cardiovascular events. Since periodontal diseases become prevalent with age, oral health screening could unnecessarily overestimate cardiovascular risk.

### 5.2. False Alarms and Overtesting

Screening tests are not perfect and will inevitably yield false positive results, indicating the risk or presence of a condition when none is actually present. Furthermore, there are very high chances that screening will detect common abnormalities that have little clinical significance yet will, in turn, trigger more testing and possible intervention. Typical examples include “pre-hypertension”, “pre-diabetes”, or OPMDs with negligible risk of malignant transformation. In one example, it has been suggested that testing HbA1c levels in routine dental practice using gingival crevicular blood “could prove to be useful to identify patients with undiagnosed diabetes” [68]. However, this screening in a dental setting would add to the (over)diagnosis of prediabetes, a common condition with no clear risk prediction path to diabetes [105] and in which the benefit of detection is often offset by its harm [106]. Similarly, the use of molecular screening biomarkers for both oral cancer and OPMDs would also present the same set of problems, with many false positive results (oral cancer is rare) and overdiagnosis (there is a large reservoir of OPMDs, mostly inconsequential [54]).

In summary, non-rational screening can lead to unnecessary anxiety, further testing, and treatment for individuals who do not have the condition. Also, while follow-up procedures can reliably confirm the presence of these abnormalities, they may still lead to unnecessary interventions for conditions that would not have posed a threat to the individual’s health.

### 5.3. Overdiagnosis and Overtreatment

Follow-up procedures following a positive screening test, even when conducted judiciously to confirm the diagnosis of individuals identified as “at risk”, can increase overdiagnosis and overtreatment through several mechanisms.

First, screening tests may signal the presence of abnormalities or early-stage conditions that would not have progressed or caused harm if left undetected. Even when these conditions are destined to become clinically evident, screening tests can detect them at an earlier stage than they would have been diagnosed clinically. While this may appear beneficial, it can artificially inflate the benefits of early detection due to the increased lead time, without necessarily improving quality of life or extending life expectancy [35].

When follow-up procedures confirm the presence of a disease or condition, individuals may still receive treatment that is more aggressive or invasive than necessary, owing to the diagnosis. This may occur due to clinical uncertainty, patient preferences, or healthcare provider biases, and can lead to unnecessary harm and burden for the individual [88]. For example, a patient who tested positive for incipient caries may undergo operative care (rather than the recommended wait-and-see or fluoride varnish). A patient diagnosed with leukoplakia may undergo repeated testing, repeat biopsy and strict follow-up, or surgery, despite the lesion not being at substantial risk of transformation.

Overall, biomarkers designed for screening must carefully balance the benefits of early detection with the risks of overtesting, overdiagnosis, and overtreatment. In this context, the development of novel molecular biomarkers should be aimed at assessing clinically significant risks, e.g., rapidly progressing or severe disease.

## 6. Conclusions

As the number of measurable parameters continues to expand exponentially, biomarker development has gained considerable attention, given its potential to benefit millions of people. However, non-rational use of biomarkers tends to simply promote further testing, regardless of the genuine utility of the tests themselves. This leads to increased testing, resulting in the detection of more abnormalities and an apparent rise in disease prevalence, which in turn fosters more concern and subsequent testing and treatment.

Here, we outlined the limitations of screening for three important oral conditions with diverse epidemiology, and we proposed a number of characteristics that should guide the development and use of novel screening biomarkers to detect the features that will inform further action while minimizing unnecessary interventions for individuals who are unlikely to benefit from them.

In conclusion, the role of screening biomarkers in oral diseases is multifaceted, and while they can provide valuable insights, they must be interpreted within the broader clinical context. The specificity and sensitivity of these markers can vary, and false positives or negatives are a possibility. Hence, their application must be guided by a thorough understanding of their capabilities and limitations. This emphasizes the need for a more logical approach to screening and biomarker development.

## Figures and Tables

**Table 2:** Examples of potential molecular biomarkers for the detection of oral cancer, periodontal disease, and dental caries in clinical settings.

Disease	Molecular Biomarkers	Refs
Oral cancer	MMP-9, IL-6, IL-8 and TNF-α; arsenic, selenium, cerium; GDF15, MMP3, AXL, MCSF, I309, CTACK; SNPs; miRNAs	[9,39,40,41,42,43,44]
Periodontal disease	IL-6, IL-1β, MIP-1α MMP-8; MMP-9; Pg, ICTP, PGE2	[45,46,47,48,49]
Dental caries	Mucin-1, histatin-5, proline-rich protein-1, lactoperoxidase, C-reactive protein, statherin, SOD, copper, zinc, chloride, alpha-amylase, carbonic anhydrase 6, proteinase-3	[50,51,52]

MMP, matrix metalloproteinase; IL, interleukin; TNF, tumor necrosis factor; SOD, superoxide dismutase; GDF, Growth/differentiation factor; MCSF, macrophage colony-stimulating factor; SNP, single-nucleotide polymorphism; MIP, macrophage inflammatory proteins.
